# Excision Repair Cross Complementation Group 1 Single Nucleotide Polymorphisms and Nivolumab in Advanced Non-Small Cell Lung Cancer

**DOI:** 10.3389/fonc.2020.01167

**Published:** 2020-09-02

**Authors:** Marco Maria Aiello, Cinzia Solinas, Matteo Santoni, Nicola Battelli, Nunzio Restuccia, Fiorenza Latteri, Sabrina Paratore, Francesco Verderame, Giuseppina Valeria Albanese, Paolo Bruzzi, Hector Josè Soto Parra

**Affiliations:** ^1^Oncology Unit, Azienda Ospedaliero Universitaria Policlinico Vittorio Emanuele, Catania, Italy; ^2^Molecular Immunology Unit, Institut Jules Bordet, Brussels, Belgium; ^3^Azienda AUSL, Regional Hospital of Aosta, Aosta, Italy; ^4^Oncology Unit, Ospedale di Macerata, Macerata, Italy; ^5^Oncology Unit, Azienda Ospedaliera Ospedali Riuniti Villa Sofia Cervello, Palermo, Italy; ^6^Clinical Epidemiology, IRCCS Azienda Ospedaliera Universitaria San Martino, IST Istituto Nazionale per la Ricerca sul Cancro, Genoa, Italy

**Keywords:** NSCLC, ERCC-1, SNP, C8092A, nivolumab, immunotherapy, PD-1, PD-L1

## Abstract

**Background:** We hypothesized that non-small cell lung cancer (NSCLC) patients with a tumor positive for single nucleotide polymorphisms (SNPs) of the Excision Repair Cross Complementation Group 1 (ERCC-1) gene could be more genetically instable and consequently more responsive to a programmed cell death-1 (PD-1) blockade.

**Methods:** We evaluated the *T19007C* and *C8092A ERCC-1* SNPs by pyrosequencing assay, on tumor specimens from two independent cohorts of patients who relapsed after one or more prior systemic treatments for advanced NSCLC and who received nivolumab (3 mg/kg intravenously every 2 weeks) as part of the Italian Expanded Access Program. We aimed to assess the outcome of enrolled subjects according to the *ERCC-1* SNPs *status*, to evaluate the role of these polymorphisms as putative biomarkers associated with a response/clinical benefit to anti-PD-1 therapies.

**Results:** Of the 45 patients included in the final analysis, 21 (47%) and 16 (36%) were positive for the *T19007C* and *C8092A* polymorphic genotype (PG), respectively. In univariate analyses, overall survival (OS) and progression free survival (PFS) were shorter in patients with the *T19007C* PG, but neither difference achieved statistical significance (*P* = 0.131 and *P* = 0.717, respectively). The presence of the *C8092A* PG was associated with a longer OS and PFS, although statistical significance was only reached for PFS (*P* = 0.112 and *P* = 0.025, respectively). These results were confirmed by multivariate analyses. The response rate was only significantly higher in patients with the *C8092A* PG vs. wild type *ERCC-1* (62 vs. 7%, *P* < 0.001).

**Conclusions:** Results from this hypothesis generating pilot study, provided suggestive evidence that a subgroup of NSCLC patients could benefit differently from nivolumab according to the *C8092A ERCC-1* SNP *status*. However, these data warrant further investigation.

## Introduction

Non-small cell lung cancer (NSCLC) is the leading cause of cancer deaths worldwide (1). In the last 30 years, survival rates have remained poor and the long-term survival is rare despite the use of standard chemotherapy (2).

Recently, immunotherapeutic strategies based on the use of monoclonal antibodies (mAbs) engaging and blocking the programmed cell death−1 (PD-1) receptor (nivolumab and pembrolizumab) or its ligand PD-L1 (atezolizumab) have shown durable clinical benefits in patients with advanced NSCLC (3–6). These drugs are called immune checkpoint blockade (ICB). Indeed, significant improvements in survival outcomes in either squamous or non-squamous NSCLC [vs. chemotherapy in second line (7–10) and in first line only for patients with high expression of PD-L1 (11)] were observed. Thus, pembrolizumab, nivolumab, and atezolizumab have become the new standard of care in these settings.

The role of PD-L1 as a predictive biomarker of response to ICB in lung cancer is still being debated. Although an increased PD-L1 expression on tumor cells is associated with better clinical outcomes in patients treated with anti PD-1/PD-L1 mAbs, its low or absent expression is not an absolute indicator of immunotherapy's lack of activity. On the contrary, a strong PD-L1 positivity does not provide the certainty of a response (7–11).

These uncertainties suggest the possibility that further biological and/or molecular features could be involved in mechanisms of response to ICB and could be exploited as additional reliable biomarkers to improve the patient's selection.

ICBs were shown to be particularly effective in NSCLC and melanoma, tumors that mainly develop after a chronic exposure to mutagens (i.e., smoke's carcinogens and ultraviolet light, respectively). It has been hypothesized that their mutational landscape may have an influence in responses to ICB.

Interestingly, Rizvi et al. demonstrated that the efficacy of pembrolizumab in NSCLC was associated with an elevated burden of neo-antigens, due to the deficiency of genes related to DNA-repair systems (12). In addition, McGranahan et al. explained that T cell immunoreactivity and sensitivity to ICB were linked to the presence of clonal neo-antigens (13).

Similarly, Le et al. observed that mismatch-repair (MMR) deficient tumors, characterized by the highest tumor mutational load, are more responsive to pembrolizumab than MMR proficient tumors (14).

The tumor mutational burden (TMB) is the number of somatic mutations detected in the tumor tissue. The phase III CheckMate 227 trial, enrolling patients with stage IV or recurrent NSCLC treated with the combination of nivolumab and the anti-Cytotoxic T Lymphocyte Antigen 4 (CTLA-4) ipilimumab (vs. chemotherapy or vs. nivolumab), showed a benefit in progression free survival (PFS) in the group treated with ICB (vs. chemotherapy) having a high TMB (≥10 mutations per megabase), irrespective of the PD-L1 expression level (15). Further, TMB can be evaluated in plasma, with this technique having the advantage of avoiding sampling bias that could be linked to difficulties in obtaining tissues from the primary tumors or from the metastatic sites and that could be affected by tumor heterogeneity. However, here the scenario becomes more complicated, since TMB assessed in plasma did not match with TMB assessed in tumor tissues, rendering further investigations necessary (16).

The excision repair cross complementation group 1 (*ERCC-1*) gene, encoding for the key enzyme of the DNA Nucleotide Excision Repair (NER) pathway, has been widely investigated in NSCLC due to its essential role in repairing platinum-DNA adducts. Its deficiency may improve the efficacy of platinum-based chemotherapy (17, 18). *ERCC-1* deficient fibroblasts were shown to spontaneously accumulate unrepaired lesions and DNA double-strand breaks, resulting in increased mutation rates and genome instability (19). These pre-clinical results were confirmed in a study with NSCLC patients where *ERCC-1* negative tumors presented a higher rate of genomic aberrations as a result of their genetic instability, while a lower number of DNA alterations were seen in *ERCC-1* positive NSCLC tumors (20).

The two most common single nucleotide polymorphisms (SNPs) of the *ERCC-1* gene, rs11615 (*T19007C*), and rs3212986 (*C8092A*) (21), are further associated with a reduced capacity of the NER pathway to repairing DNA damage (22–24). Specifically, the *T19007C* SNP is set in the codon 118 of the *ERCC-1* gene and determines a slower mRNA translation, and is associated with a better response to a platinum-based chemotherapy in lung tumors (25, 26). In contrast, the *C8092A* SNP, located in the 3′-untranslated region of the *ERCC-1* gene, reduces mRNA stability, and seems to be related to increased overall survival (OS) and grade 3–4 toxicity in patients with advanced NSCLC treated with platinum compounds (27–29).

Based on these data, we hypothesize that NSCLC patients with a tumor positive for *ERCC-1* gene SNPs could be more genetically instable, could present higher mutational load, and consequently could be more responsive to PD-1 ICB when compared to subjects negative for these genetic alterations.

In this regard, the Italian Expanded Access Program (EAP) of nivolumab in NSCLC represented an important opportunity to test our hypothesis, and we started an exploratory analysis evaluating the *T19007C* and *C8092A ERCC-1* gene polymorphisms from two independent cohorts of patients enrolled in this study.

Here, we report the outcomes of NSCLC patients according to the *ERCC-1* SNPs *status* and attempt to evaluate the role of these genetic alterations as possible future, hypothetical, promising biomarkers of response/clinical benefit to nivolumab.

## Materials and Methods

### Patients

While nivolumab was evaluated by the European Medicines Agency (EMA) and negotiations with the Italian Ministry of Health were ongoing, an Italian EAP was initiated from July 2015 to April 2016, allowing advanced NSCLC patients to access this treatment before it became commercially available.

Eligible patients were 18 years of age or older with histologically or cytologically proven stage IIIB or IV NSCLC that had progressed or recurred during or after one or more prior systemic treatments for the advanced or metastatic disease. Each patient was required to have at baseline: (1) an Eastern Cooperative Oncology Group (ECOG) Performance Status (PS) ≤ 2; (2) resolution of all adverse events (AEs) to grade one according to the Common Terminology Criteria for Adverse Events (CTCAE) v. 4.3; (3) an adequate organ function with life expectancy > 6 weeks; and (4) completion of prior chemotherapy, therapy with tyrosine-kinase inhibitors or palliative radiotherapy ≤2 weeks before starting nivolumab.

Active autoimmune diseases, carcinomatous meningitis, symptomatic interstitial lung disease, systemic immunosuppression, or prior therapy with T-cell stimulation and other ICB were considered key exclusion criteria.

Furthermore, to be included in the present analysis, it was mandatory that patients had: (1) a complete clinical and radiological assessment at baseline with at least one measurable lesion per RECIST v.1.1; (2) received ≥1 dose of nivolumab; and (3) provided a sufficient amount of tumor tissue for the analysis of *ERCC1* gene SNPs: in formalin-fixed paraffin-embedded (FFPE) tissues or cytological samples, a minimum of 15% of tumor cells should be present to avoid false-negative results.

All patients signed a written informed consent form that was based on the principles of the Declaration of Helsinki for the use of their data for research purposes.

### Study Design

Nivolumab was available on physician request through the EAP and was administered at the dose of 3 mg/kg intravenously every 2 weeks for 24 months or until disease progression, unacceptable toxicity, or withdrawal of consent. Treatment delay due to nivolumab-related AEs was allowed, while dose escalation or reduction was not permitted. It was possible to continue the treatment beyond disease progression: (1) according to the investigator's choice; (2) if the patient tolerated the study drug well; (3) in the presence of a stable PS; (4) if the patient was achieving clinical benefit in the absence of a rapid disease progression; and (5) if the continuation of the treatment would not delay any intervention to prevent serious complications related to disease progression.

The following endpoints were evaluated: OS; PFS and objective response rate (ORR) according to RECIST v.1.1. These outcomes were assessed in different subgroups based on the baseline presence or absence of the *T19007C* and *C8092A ERCC-1* gene SNPs.

Patients were continuously followed for survival while they were receiving the study drugs and every 3 months after the discontinuation of the treatment. Those without documented clinical or radiographic disease progression were censored on the date of last follow-up. The data cut-off was 14th November 2016.

All clinical efficacy data of patients enrolled in the EAP and presented here, were prospectively collected during the program using a dedicated chart.

The study protocol was reviewed and approved by the Ethics Committee “Catania 1” of the Azienda Ospedaliero Universitaria Policlinico Vittorio Emanuele, Catania, Italy, as appropriate, and was conducted in accordance with Good Clinical Practice guidelines, as defined by the International Conference on Harmonization.

### Genotyping of the *ERCC-1* Gene Polymorphisms

Two FFPE tissue sections (10 μm thickness) or a sufficient volume of the cytological sample (FNA/bronchial-brushing)—that was dependent on the percentage of tumor cells present in the sample—were obtained from all enrolled patients. Tumor cells were dissected, and genomic DNA was extracted by QIAmp FFPE tissue and automatic extractor (Qiagen) following the manufacturer's instructions. DNA quality control and yield were assessed by spectrophotometry using a Nanodrop machine (Thermo Scientific).

Genotyping of two selected *ERCC-1* gene variants, *T19007C* (SNP reference n° rs11615, hotspot mutation p.Asn118Asn) and *C8092A* (SNP reference n° rs3212986, hotspot mutation p.Gln506Lys), was performed by pyrosequencing (PyroMark 24 system, Qiagen) as per the manufacturer's protocol. The specific primer was designed by studying the gene sequence of the human gene *ERCC-1* present in GenBank and using the AssaY Design Software, Version 1.0.1: forward biotinylated primer 5′-TCCCGGGGGCAGACTACA-3′, reverse primer 5′-AGTCAGGAAAGCCGGATGC-3′. 10–100 ng of tumor DNA and control DNA (commercially available, Qiagen) were amplified by polymerase chain reaction (PCR) with the following reaction conditions: 95°C-denaturation for 4 min, 45 cycles of 95°C for 30 s, 57°C for 30 s, and 72°C for 30 s, with a final extension of 72°C for 3 min. The biotinylated PCR products combined with specific primers (GGACAAGCAGCGGAA) were then immobilized on streptavidin-coated Sepharose beads (GE Healthcare), and the single-stranded DNA templates were analyzed by PyroMark Q24 (Qiagen).

The identification of *ERCC-1* polymorphism was performed by comparing the DNA sequence from tumor samples with the sequence of the DNA control using PyroMark Q24 software. Robust and sensitive sequence data were obtained with a detection limit of the mutant allele of 5%.

### Statistical Analysis

The analyses were primarily aimed at evaluating the independent prognostic role of the two selected *ERCC-1* gene SNPs, *T19007C* and *C8092A*, in patients treated with nivolumab. To this aim, OS (computed from the first day of nivolumab therapy to the day of death or last follow-up) and PFS (computed from the first day of nivolumab therapy to the day of progression or death or to the day of last follow-up, whichever first) were compared in patients with and without each one of the two polymorphisms with standard univariate techniques, estimating Kaplan Meier survival curves and comparing them with the log-rank test. To rule out a possible confounding effect of other variables known to be associated with prognosis and to assess the independent and combined effect of the two variants when considered together, several multivariate models were fitted to OS and PFS data, with the two gene variants and histology (squamous vs. non-squamous), age (< 65 vs. >65 years), ECOG PS (0 vs. 1–2), gender (male vs. female), and number of prior systemic regimens (1 vs. 2 vs. >3) as covariates. The final models were obtained by means of a backward procedure based on the likelihood ratio test, starting from the full model, which included all the above-mentioned covariates. Due to the small numbers involved, which precluded convergence in the estimation of the coefficients of the model, an interaction term aimed at assessing the (negative or positive) synergism between the two gene variants could not be included in the full model. As a consequence, the presence of synergism was evaluated by comparing the log-likelihood of two reduced models that included only the two gene variants, with and without the interaction factor between them. The association between the gene variants and objective response (OR) was assessed in a similar fashion, first in univariate analyses by comparing the proportion of responders in patients with and without the mutation with standard chi-square (χ^2^) or Fisher's test, as appropriate, and then fitting a logistic regression model with the OR as the dependent variable and with the above-mentioned variables as covariates.

All *P*-values are 2-sided. However, in light of the exploratory nature of this investigation, *P*-values must be considered with caution, both when they achieve formal statistical significance (*P* < 0.05), due to a multiplicity of problems, and when they do not, due to the small sample size and associated low power.

## Results

### Patients

From July 2015 until April 2016—the duration of the EAP in Italy—treatment with nivolumab in the EAP was requested and obtained for 55 and 45 patients at the Oncology Units of Policlinico–Vittorio Emanuele Hospital, Catania, Italy and Villa Sofia–Cervello Hospital, Palermo, Italy, respectively.

Of the 55 patients enrolled in the EAP and treated with nivolumab at the Oncology Unit of Policlinico–Vittorio Emanuele Hospital, 24 subjects met all criteria to be included in the present analysis and composed the first cohort. Reasons for ineligibility were insufficient tumor tissue for the analysis of the *ERCC-1* gene SNPs (*N* = 25 patients) and lack of measurable lesions per RECIST v.1.1 at baseline (*N* = 6 patients).

The second cohort was represented by a group of 21 out of 45 subjects fulfilling all criteria for our analysis. They were enrolled in the EAP and treated with nivolumab at the Oncology Unit of Villa Sofia—Cervello Hospital. Also, in this case, the main exclusion criteria were the unavailability of adequate tumor tissue for the SNPs analysis (*N* = 20 patients) followed by the absence of radiological target lesions at baseline (*N* = 4 patients).

The data of the two cohorts were pooled, and the analysis was performed on a total of 45 patients. Baseline clinical and pathological characteristics were similar in both cohorts and are summarized in Table 1. The median age at diagnosis was 64 years. Most patients had an ECOG PS of 0 (73%), a stage IV cancer (82%) and were current or former smokers (89%). The most frequent histological type was adenocarcinoma (76%) and *Epidermal Growth Factor Receptor* (*EGFR*) activating mutations were found in 9% of patients while no *Anaplastic Lymphoma Kinase* (*ALK*) translocations were detected. All patients were treated with one or more previous chemotherapy regimens.

**Table 1:** Patients characteristics.

**Characteristics**	**Patients (*N* = 45)**
Median age (range)–years	64.0 (38.0–80.0)
Sex–*N* (%)	
Female	8 (18)
Male	37 (82)
ECOG performance status–*N* (%)	
0	33 (73)
1	9 (20)
2	3 (7)
Disease stage at diagnosis–*N* (%)	
IIIB	8 (18)
IV	37 (82)
Smoking status–*N* (%)	
Current or former smoker	40 (89)
Never smoked	5 (11)
Histology–*N* (%)	
Non-squamous NSCLC	34 (76)
Squamous NSCLC	11 (24)
Positive *EGFR* mutation status–*N* (%)	4 (9)
Positive *ALK* translocation status–*N* (%)	0 (0)
No. of prior systemic regimens–*N* (%)	
1	13 (29)
2	18 (40)
≥3	14 (31)
Type of prior systemic therapy–*N* (%)	
Platinum based therapy	45 (100)
EGFR tyrosine kinase inhibitor	4 (9)
Best response to most recent systemic regimen–*N* (%)	
Complete or partial response	19 (42)
Stable disease	15 (33)
Progressive disease	11 (25)

All patients received at least one dose of the study drug and a mean of 12 doses (range, 1–28) were administered. At the time of data cut-off, 12 subjects (27%) were still under nivolumab treatment and the median follow-up for OS was 11.0 months [Interquartile range (IQR) = 8.9–12.7 months], while the median follow-up for PFS was 9.7 months (IQR = 8.7–11.9 months).

### The Frequencies of Genotypes

Allele quantification by pyrosequencing assay at the *T19007C* and *C8092A ERCC-1* polymorphisms was performed on 45 NSCLC specimens. No discrepancies in the samples, analyzed in duplicate for quality control, were observed, and all the genotyping data were included in the final analysis. The allelic frequencies observed are shown in Table 2. Alleles of both SNPs were in Hardy-Weinberg equilibrium (HWE). The Minor allele frequency (MAF) was 0.22 for *rs11615*, 0.26, and for *rs3212986*. MAFs were deviated from those publicly available (of around 0.36 and 0.29, respectively) at the NCBI SNP Database (www.ncbi.nlm.nih.gov/snp/), likely due to the small number of analyzed subjects. The distribution of genotypic frequencies is reported in Table 3: TT (reference genotype), CT (heterozygous variant), and CC (homozygous variant) genotypes for *T19007C* polymorphism were 53% (*N* = 24), 42% (*N* = 19), and 5% (*N* = 2), respectively, while the frequencies of CC (reference genotype), AC (heterozygous variant), and AA (homozygous variant) genotypes for the *C8092A* SNP were 64% (*N* = 29), 27% (*N* = 12), and 9% (*N* = 4), respectively.

**Table 2:** Allelic frequencies of ERCC-1 gene single nucleotide polymorphisms.

**SNP**	**Major/Minor**	**Allelic**				**HWE**
	**allele**	**frequencies**				
T19007C	T/C	T	0.74	C	0.26	0.54
C8092A	C/A	C	0.78	A	0.22	2.34

**Table 3:** Genotyping of the *ERCC-1* gene single nucleotide polymorphisms.

**Genotype**	***N* (%)**
***T19007C***	
TT (reference genotype)	24 (53)
CT (heterozygous variant)	19 (42)
CC (homozygous variant)	2 (5)
CT + CC (polymorphic genotype)	21 (47)
***C8092A***	
CC (reference genotype)	29 (64)
AC (heterozygous variant)	12 (27)
AA (homozygous variant)	4 (9)
AC + AA (polymorphic genotype)	16 (36)
**Both** ***T19007C*** **+** ***C8092A*** PG^*^	11 (24)
**Only** ***T19007C*** **PG**	10 (22)
**Only** ***C8092A*** **PG**	5 (11)
**No** ***T19007C*** **PG nor** ***C8092A*** **PG**	19 (42)

* *Odds Ratio = 4.18 (95% CI, 1.13–15.42); P = 0.027*.

Allelic frequencies and relative excess of heterozygosity was determined to check compatibility of the genotype frequencies with HWE. Thus, the *P*-value from the exact HWE lack was calculated by a goodness-of-fit χ^2^-test.

In our analysis, we decided to group together both the heterozygous and homozygous variants of the given polymorphism as “polymorphic genotype” (PG) [21 PGs out of 45 patients (47%) for *T19007C* SNP and 16 PGs out of 45 patients (36%) for *C8092A* SNP], while we referred to the TT variant for *T19007C* SNP and the CC variant for *C8092A* SNP as reference genotypes (RGs) since they are considered to be the “reference variants” by convention because they generate the more frequent codon sequence encoding the amino-acid.

Overall, 11 (25%) out of 45 patients were simultaneously positive for both *T19007C* and *C8092A* PGs, while we found only 10 (22%) and five (11%) subjects with an exclusive *T19007C* or *C8092A* PG, respectively. Furthermore, 19 (42%) out of 45 patients presented none of the two PGs.

A statistically significant positive association between the two polymorphisms was observed [Odds Ratio (OR) = 4.18 (95% CI, 1.13–15.42); *P* = 0.027, Table 3.

### Overall Survival

At the time of data cut-off, 18 patients (40%) had died. Median OS had not been reached, and 1-year OS was 75.4% (±6.4%).

**Figure 1:**
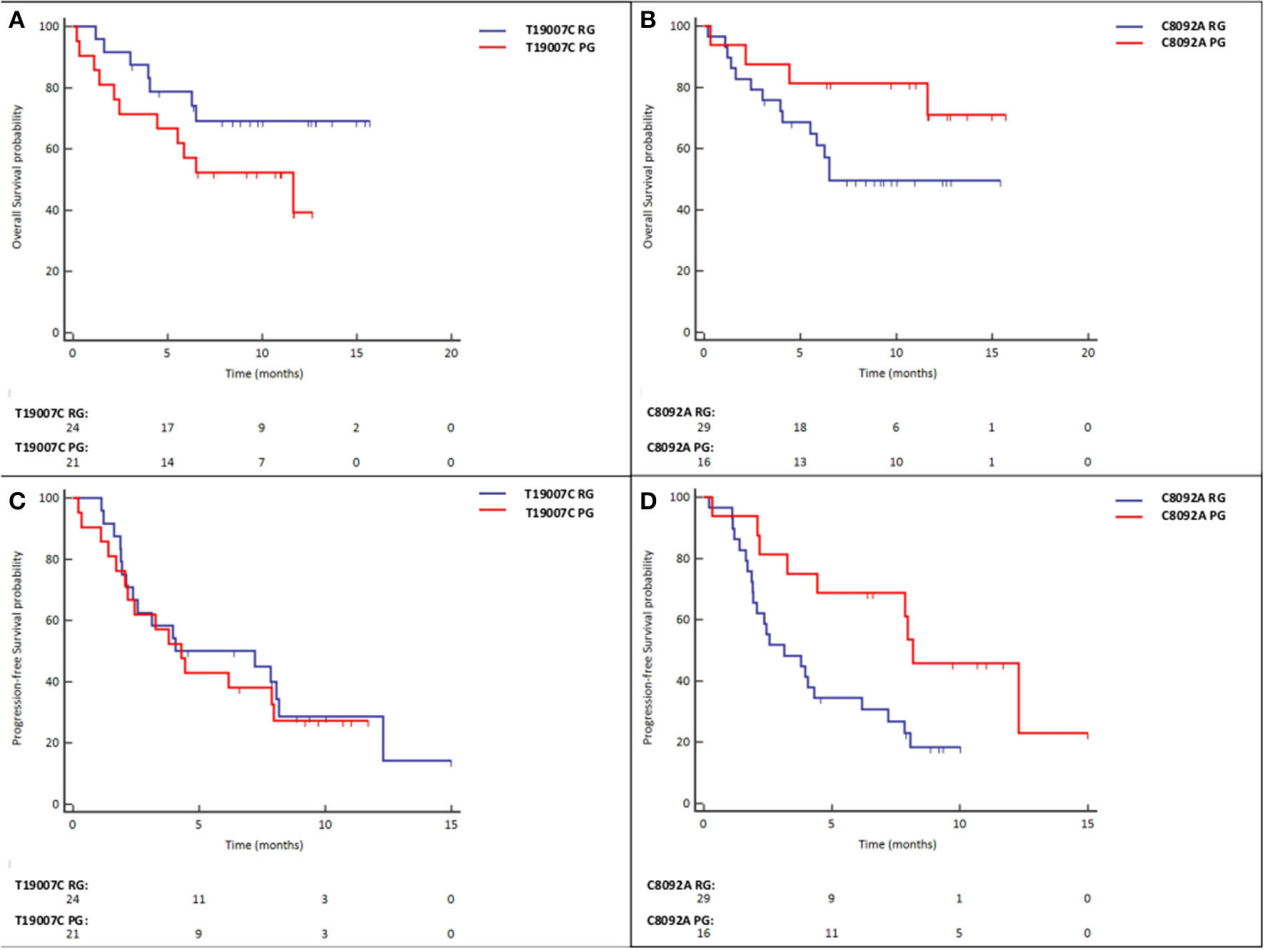
**(A)** Overall Survival in *T19007C* RG vs. *T19007C* PG patients. **(B)** Overall Survival in *C8092A* RG vs. *C8092A* PG patients. **(C)** Progression-free Survival in *T19007C* RG vs. *T19007C* PG patients. **(D)** Progression-free Survival in *C8092A* RG vs. *C8092A* PG patients.

In univariate analyses, a shorter OS was seen in patients with the *T19007C* PG [7.9 vs. 12.1 months, Hazard Ratio (HR) for death = 2.08 (95% CI, 0.80–5.37); *P* = 0,131, Figure 1A] while the *C8092A* PG was associated with a longer OS [12.8 vs. 9.5 months, HR for death = 0.40 (95% CI, 0.13–1.24); *P* = 0.112, Figure 1B], but neither difference achieved statistical significance.

These results were confirmed in multivariate analyses (Table 4), in which only three variables were retained in the final model: gender (*P* = 0.005), presence of the *T19007C* PG [HR = 4.90 (95% CI, 1.69–14.22); *P*=0.004] and presence of the *C8092A* PG [HR = 0.19 (95% CI, 0.06–0.62); *P* = 0.006].

**Table 4:** Univariate and Multivariate analyses for OS, PFS, and ORR.

**Univariate analyses**				Multivariate analyses^*^		
**OS**	**HR**	**95% CI**	***P***	**HR**	**95% CI**	***P***
*T19007C* PG	2.08	0.80–5.37	0.131	4.90	1.69–14.22	0.004
*C8092A* PG	0.40	0.13–1.24	0.112	0.19	0.06–0.62	0.006
				*P* for interaction: NA		
**PFS**	**HR**	**95% CI**	***P***	**HR**	**95% CI**	***P***
*T19007C* PG	1.14	0.56–2.30	0.717	2.47	1.16–5.26	0.018
*C8092A* PG	0.39	0.17–0.89	0.025	0.17	0.07–0.44	<0.001
				*P* for interaction: NS		
**ORR**	**Odds ratio**	**95% CI**	***P***	**Odds ratio**	**95% CI**	***P***
*T19007C* PG	1.20	0.32–4.51	0.787	0.08	0.004–1.59	0.099
*C8092A* PG	22.5	3.88–130.41	<0.001	301.13	8.21–11045.48	0.002
				*P* for interaction: NS		

* *For OS and PS, a Cox Proportional Hazard Model was fitted to the data starting with the following covariates: T19007C PG (Positive vs. Negative), C8092A PG (Positive vs. Negative), Histology (Squamous vs. Non-Squamous), Age (≤ 65 vs. >65 years), ECOG PS (0 vs. 1–2), Gender (Male vs. Female), Number of prior systemic regimens (1 vs. 2 vs. >3). In both final models (OS and PFS) only T19007C PG, C8092A PG and gender were retained (P < 0.05)*.

*For ORR, a logistic regression model was used with objective response as the dependent variable and same covariates used in the PFS and OS models. Only T19007C PG, C8092A PG, and age were retained in the final model (P < 0.05)*.

*The interaction between T19007C PG and C8092A PG was assessed within the same 3 final models, by evaluating the change in the log-likelihood of the model associated with the introduction of the interaction term. However, for OS, the p for interaction could not be estimated because of failure of model convergence*.

No significant interaction between the two polymorphisms was observed (P for interaction = 0.218).

### Progression Free Survival

At the time of data cut-off, 32 patients (71%) had progressed, with a median PFS of 4.3 months (IQR: 1.22–73.8 months).

In univariate analyses, the presence of a *T19007C* PG was not associated with a longer PFS [5.6 vs. 6.7 months, HR for disease progression or death = 1.14 (95% CI, 0.56–2.30); *P* = 0.717, Figure 1C], whereas the C8092A PG was associated with a significantly higher PFS [8.9 vs. 4.5 months, HR for disease progression or death = 0.39 (95% CI, 0.17–0.89); *P* = 0.025, Figure 1D].

In multivariate analyses (Table 4), again the only variables associated with PFS were gender (*P* = 0.001), presence of the *T19007C* PG [HR = 2.47 (95% CI, 1.6–5.26); *P* = 0.018] and presence of the *C8092A* PG [HR = 0.17 (95% CI, 0.07–0.44); *P* < 0.001].

No significant interaction between the two polymorphisms was observed (P for interaction = 0.818).

### Objective Response

Twelve patients had achieved an OR (27%) with one complete response (CR). Eight patients (18%) presented stable disease (SD) and 25 (55%) had disease progression (PD) (see Table 5).

**Table 5:** Response rates.

	**Type of response**						
**Genotype**	**SD**	**PD**	**CR**	**PR**	**SD + PD**	**ORR (CR + PR)**	
All patients (*N* = 45)	8 (18%)	25 (55%)	1 (2%)	11 (25%)	33 (73%)	12 (27%)	
*T19007C* RG (*N* = 24)	5 (21%)	13 (54%)	0 (0%)	6 (25%)	18 (75%)	6 (25%)	*P* = 0.787
*T19007C* PG (*N* = 21)	3 (14%)	12 (57%)	1 (5%)	5 (24%)	15 (71%)	6 (29%)	
*C8092A* RG (*N* = 29)	7 (24%)	20 (69%)	0 (0%)	2 (7%)	27 (93%)	2 (7%)	*P* < 0.001
*C8092A* PG (*N* = 16)	1 (6%)	5 (32%)	1 (6%)	9 (56%)	6 (38%)	10 (62%)	

No association was seen between response rate (RR) and the *T19007C* PG. Conversely, the *C8092A* PG was significantly associated with OR, as ORR was 62% for patients with PG and 7% for the *C8092A* reference genotype's group (*P* < 0.001–Table 5). When a multivariable logistic regression analysis was fitted to the data with OR as the dependent variable and the above-mentioned variable as covariates, only age (*P* = 0.004) and *C8092A* (*P* < 0.0001) were significantly associated with RR, while an association of borderline statistical significance (*P* = 0.099) with the *T19007C* genotype was observed (Table 4). No significant interaction between the two polymorphisms was seen (*P* = 0.567).

## Discussion

Our analysis showed a marked and statistically significant increase in both ORR and PFS in patients with a *C8092A* PG of the *ERCC-1* gene when compared to those with a *C8092A* reference genotype. The improvement in OS was comparable to that observed in PFS, although the smaller number of events was associated with a much larger CI and precluded the attainment of a statistical significance.

Data from our study support the hypothesis that NSCLC patients with tumors positive for the *C8092A* SNP could benefit differently from nivolumab than subjects without this genetic alteration. Conversely the presence of the *T19007C* SNP does not seem to have a role in patients treated with the study drug or may even be detrimental.

This difference could be explained by hypothesizing that the mRNA instability linked to the *C8092A* SNP, results in a decreased synthesis of the ERCC-1 enzyme. This leads to an accumulation of the DNA damage, rendering the tumor more immunogenic and potentially more responsive to nivolumab. In contrast the *T19007C* SNP seems to affect only the speed of mRNA translation, determining a slower but not reduced protein expression that improves response to alkylating agents, which act very rapidly on tumor cells, but probably has no effect on the tumor immunogenicity since the ERCC-1 enzyme is still slowly produced.

However, we should be cautious with the interpretation of these results, considering the small cohort analyzed and the retrospective nature of the analysis. Indeed, conclusions were drawn based on results obtained in 16 patients harboring the *C8092A* SNP variant. Further, the lack of a control group does not allow us to evaluate whether the SNPs perhaps have a prognostic or predictive significance in patients treated with PD-1 ICB.

In this era of precision medicine and of targeted therapies, the identification of reliable biomarkers to select patients who are more likely to benefit from potentially toxic ICB (30–34) is crucial and still an open challenge in oncology, also considering the cost of drugs and their impact on the health care systems (35).

PD-L1 expression on tumor cells evaluated by immunohistochemistry (IHC) was the first identified as a potential indicator of benefit and a “logical” biomarker for the prediction of response to PD-1/PD-L1 ICB in lung cancer. Furhter, PD-L1 was largely investigated in a variety of neoplastic diseases with conflicting results (36).

Nowadays, PD-L1 expression (at different cut-offs) is determined only to select metastatic NSCLC candidates to be treated with pembrolizumab both in first and second line, since a clear association between PD-L1 positive tumors and an enhanced clinical benefit with this anti PD-1 antibody was detected in pivotal studies (9, 11).

Conversely, data from clinical trials of nivolumab and atezolizumab showed that the PD-L1 expression level is also an important, but not definitive, predictive biomarker of response to these two drugs. Indeed, they are both approved in the second line setting regardless of PD-L1 positivity (7, 8, 10).

The diverging results of PD-L1 expression emerged in clinical trials with anti PD-1/PD-L1 ICB and are strictly dependent on the intrinsic PD-L1 biology [e.g., its expression can be constitutive or inducible after cytokine exposure (37)], and methods used for PD-L1 IHC testing. Indeed, PD-L1 is an inducible protein whose expression is subject to temporal and spatial variation on tumor cells and could be affected by prior treatments (38, 39). Different from classical “binary” (positive or negative) biomarkers in NSCLC, such as *EGFR* activating mutations and *ALK* rearrangements, the expression of PD-L1 is a continuous variable, starting from zero through to high levels, and the use of thresholds to define if the IHC assay is positive or negative is artificial, generating the illusion of a binary system. All these variables can probably be justified because some PD-L1 negative subjects respond to ICB and, conversely, a subset of PD-L1 positive patients fail to achieve a clinical benefit from ICB. Moreover, the PD-L1 IHC assay is affected by a multitude of technical issues (e.g., companion tests are not equivalent in terms of antibodies and cut-offs; the way to interpret it pathologically, whether on tumor cells or immune cells, is diverse) with difficulties in interpretation and standardization of results across studies (40).

In the present study, PD-L1 status has not been initially assessed because its level of expression is not routinely used in this setting and was not necessary for the enrollment in the EAP. Further, for most of the patients PD-L1 was not evaluable due to the paucity of histological material.

Several biomarkers and combined strategies are currently being investigated (41, 42), but none of these have given good results. Among them the TMB (15, 16, 43) and the mutations in the DNA repair pathway (14, 44) were shown to identify patients that benefit from ICB. However, even if the analysis of the tumor mutational profile has recently become much easier, thanks to the improvements and availability of the DNA sequencing techniques, examining genetic signatures in daily clinical practice is very challenging because of costs, and difficulties in reproducibility.

As outlined in the introduction, the presence of microsatellite instability, high (MSI-H) or MMR deficiency was in response to PD-1 ICB (12). Afterwards a variety of studies provided consistent combined data from five disease-specific pembrolizumab clinical trials (KEYNOTE-016, KEYNOTE-164, KEYNOTE-012, KEYNOTE-028, and KEYNOTE-158). On May 2017, the United States Food and Drug Administration (FDA) granted accelerated approval for pembrolizumab in patients with unresectable or metastatic solid tumors that have MSI-H or MMR deficiency. This was the first time that a medicine agency approved a cancer treatment based on a common biomarker rather than the anatomical site where the tumor originated and/or the histotype. NSCLC patients were not included in these trials, probably because MMR defects are infrequent and do not play a crucial role in the biology of lung carcinomas.

The strength of our study is that it employed a novel potential biomarker that is based exclusively on a genetic *status* (as for tumors with MSI-H or MMR deficiency), which could be very promising to be investigated as a prognostic/predictive factor for advanced NSCLC patients treated with nivolumab. Advantages of the use of *C8092A* PG would be: (1) binary-type biomarker (which does not generate confusion, as happens with continuous variables and their thresholds of positivity), (2) low costs, (3) better reproducibility and, (4) ease of execution with respect to TMB analysis.

However, due to the exploratory nature of our investigation, the main limits of our analysis are the small sample size with associated low statistical power and the unavailability of a control group receiving a different therapy. Indeed, it was not possible to confirm that this hypothetical “predictive” role is present only in patients treated with ICB, which would imply that this gene variant predicts its efficacy (=the underlying hypothesis in this study). Nevertheless, the observation of an association between the *C8092A* PG and OS, PFS and responses would have provided strong support for this hypothesis.

In conclusion, these type of hypothesis generating studies only provide suggestive evidence to be confirmed in larger prospective studies. However, the general consistency of the results and the strength of the associations observed suggest that a confirmatory study is warranted.

## Data Availability Statement

The datasets presented in this study can be found in online repositories. The names of the repository/repositories and accession number(s) can be found below: [https://www.ncbi.nlm.nih.gov/gene/2067] and NCBI Reference Sequence: NC_000019.10.

## Ethics Statement

The studies involving human participants were reviewed and approved by Ethic committee “Catania 1” of University Hospital Polyclinic Vittorio Emanuele Catania, Italy. The patients/participants provided their written informed consent to participate in this study.

## Author Contributions

MA and HS: conceptualization and project administration. PB: data curation, methodology, and software. SP and PB: formal analysis and validation. MA, NR, FV, GA, and HS: investigation. NR, FV, GA, and HS: resources. MA, PB, and HS: supervision. MA, FL, SP, PB, and HS: writing – original draft. CS, MS, NB, FL, and HS: writing – review & editing. All authors: contributed to the article and approved the submitted version.

## Conflict of Interest

The authors declare that the research was conducted in the absence of any commercial or financial relationships that could be construed as a potential conflict of interest.
